# Evaluation of the rapid Idylla *IDH1-2* mutation assay in FFPE glioma samples

**DOI:** 10.1186/s13000-024-01492-3

**Published:** 2024-05-25

**Authors:** James P. Solomon, Carlos Munoz-Zuluaga, Cheyanne Slocum, Alicia Dillard, Lin Cong, Jiajing Wang, Neal Lindeman, Michael Kluk, Benjamin Liechty, David Pisapia, Hanna Rennert, Priya D. Velu

**Affiliations:** 1 Department of Pathology and Laboratory Medicine, Weill Cornell Medicine, 1300 York Avenue Suite C-302, New York, NY 10065 USA; 2 NewYork-Presbyterian Hospital, 525 East 68th Street Suite F-540, New York, NY 10065 USA

**Keywords:** Glioma, IDH1, IDH2, Idylla

## Abstract

*IDH1* and *IDH2* mutational status is a critical biomarker with diagnostic, prognostic, and treatment implications in glioma. Although *IDH1* p.R132H-specific immunohistochemistry is available, it is unable to identify other mutations in *IDH1/2*. Next-generation sequencing can accurately determine *IDH1/2* mutational status but suffers from long turnaround time when urgent treatment planning and initiation is medically necessary. The Idylla assay can detect *IDH1/2* mutational status from unstained formalin-fixed paraffin-embedded (FFPE) slides in as little as a few hours. In a clinical validation, we demonstrate clinical accuracy of 97% compared to next-generation sequencing. Sensitivity studies demonstrated a limit of detection of 2.5-5% variant allele frequency, even at DNA inputs below the manufacturer’s recommended threshold. Overall, the assay is an effective and accurate method for rapid determination of *IDH1/2* mutational status.

**Supplementary Information:**

The online version contains supplementary material available at 10.1186/s13000-024-01492-3.

## Introduction

The *IDH1* and *IDH2* genes encode isoforms of isocitrate dehydrogenase enzymes located in the cytosol and mitochondria, respectively, and perform oxidative carboxylation of isocitrate to a-ketoglutarate (a-KG) with the attendant reduction of NADP + to NADPH. Pathogenic missense variants concentrate in codon 132 of *IDH1* and codons 140 or 172 of *IDH2* and result not only in loss of wild-type *IDH1/2* catalytic activity, but also gain of function through reduction of a-KG to 2-hydroxyglutarate (2-HG) with consumption of NADPH. The accumulation of 2-HG in cells competitively inhibits enzymes reliant on a-KG, including those involved in the tricarboxylic acid cycle important for cellular metabolism and histone and DNA demethylases crucial for epigenetic regulation. Decreased NADPH levels result in diminished reserves of antioxidants like glutathione that protect the cell against oxidative damage and of deoxynucleotides used in repair of DNA damage [1–3].

Adult-type diffuse gliomas are the most common primary malignant brain tumors, and over 30% have a mutation in *IDH1* or *IDH2.* This is a critical prognostic marker, associated with significantly longer overall and progression-free survival compared to *IDH1/2*-wildtype adult-type diffuse gliomas. Over 90% of *IDH1/2* mutant gliomas harbor the *IDH1* p.R132H alteration while *IDH1* p.R132C/G/L/S and *IDH2* p.R172G/K/M are more rarely observed [4]. The rapid detection of *IDH1/2* mutations is not only diagnostically and prognostically important but also impacts treatment decisions, especially as clinical trials of IDH inhibitors such as ivosedinib and vorasidenib show promise at slowing disease progression in lower WHO CNS grade disease [5].

Current standard-of-care testing for *IDH1/2* alterations at our institution consists of an initial immunohistochemistry (IHC) test to detect the protein produced by the *IDH1* p.R132H alteration followed by a next-generation sequencing (NGS) panel that includes the *IDH1* and *IDH2* genes. While the IHC can provide relatively rapid results within 1–2 days of ordering, it is limited to one alteration, and while the NGS panel covers both genes entirely, the turnaround time (TAT) can reach over two weeks.

The Idylla IDH1-2 Mutation Assay Kit is a sample-to-result, cartridge-based real-time PCR assay that qualitatively detects five alterations resulting in five codon changes in *IDH1* (p.R132C/H/G/S/L) and ten alterations resulting in nine codon changes in *IDH2* (p.R140Q/L/G/W and p.R172K/M/G/S/W). Advantages of the assay include minimal hands-on time, rapid TAT, and use of FFPE tissue as direct input. Here we evaluated test performance characteristics of the Idylla IDH1-2 assay using glioma FFPE tissue samples, which can often have limited tissue and thus low DNA input.

## Materials and methods

This retrospective clinical validation study was approved by the Weill Cornell Medicine Institutional Review Board.

### Clinical cohort, samples, and controls

Clinical FFPE specimens were obtained from patients treated at NewYork-Presbyterian Hospital (NYPH) campuses between 2018 and 2023 that had a diagnosis of glioma by histology and corresponding immunohistochemistry (IHC) and molecular testing. For cartridge input, we used 1–5 unstained FFPE tissue sections (3–4 μm thick) per case. The optimal number of unstained sections to test was determined through microscopic examination of tissue and tumor quantity on an H&E slide cut serially following the unstained sections. Reference standard DNA for *IDH1* p.R132H (HD677), *IDH2* p.R172K (HD680), and wild-type *IDH1/2* (HD678, HD681) from Horizon Discovery (Waterbeach, UK) was used for limit of detection (LOD) studies at variant allele fraction (VAF) 2.5%, 5% and 7% with 10 ng, 20 ng, and 50 ng DNA inputs. Residual extracted DNA from peripheral whole blood of three patients with hematologic malignancy (*IDH1* p.R132H with VAF 46.2%, *IDH2* p.R140K with VAF 49.7%, *IDH1/2* wild-type) was tested at 200 ng, 100 ng, and 50 ng in duplicate. DNA was extracted from FFPE slides using the Maxwell 16 FFPE Plus LEV DNA purification Kit (Promega, Madison, WI, USA) with 30 μl elution volume and DNA quantitation was performed using the Nanodrop (Thermo Fisher Scientific, Waltham, MA, USA).

### Immunohistochemistry

Diagnostic IHC to determine the presence of IDH1 protein with p.R132H alteration was performed on all glioma cases as part of routine clinical evaluation using the anti-IDH1 R132H (Hu) from Mouse (H09) DIA-H09 monoclonal antibody from Dianova (Geneva, Switzerland), as previously described [6]. Strong cytoplasmic staining in tumor cells indicated the presence of mutant IDH1 protein with the p.R132H alteration.

### Next-generation sequencing

All samples had next-generation sequencing (NGS) performed as part of routine clinical evaluation by the Oncomine Comprehensive Assay v2 on the Ion Torrent platform by Thermo Fisher Scientific (Waltham, Massachusetts, USA) as previously described [6]. This assay has a limit of detection of 3% variant allele fraction and covers 143 cancer-related genes, including *IDH1* p.R132 and *IDH2* p.R140 and p.R172.

### Idylla IDH1-2 mutation assay

The Idylla *IDH1/2* PCR assay by Biocartis (Mechelin, Belgium) rapidly detects 15 of the most common *IDH1/2* mutations in acute myeloid leukemia and diffuse gliomas (*IDH1* R132C/H/G/S/L, *IDH2* R140Q/L/G/W, and *IDH2* R172K/M/G/S/W) with a run time of 1.5 h (Supplementary Table 1). The universal single-use cartridge contains all necessary generic reagents including liquid reagents for nucleic acid extraction and lyophilized reagents for qPCR reactions to test one sample at a time. The single-use vial contains all assay-specific qPCR reagents including allele-specific primers and probes for detection of *IDH1/2* alterations. Separate vials are also available for other Idylla assays, such as *EGFR*, that can be used with the same cartridge system. When a sample is ready to be tested, 50 μl of vial content and sample is added to the lysis pad at the bottom of the cartridge opening. The sample may consist of 10–50 μl of liquid (extracted DNA, direct blood or bone marrow) or up to five unstained paraffin-embedded sections sandwiched between two filter papers wetted with nuclease-free water. All processing steps happen within the cartridge and all components are only compatible with the Idylla system and reagents. The cartridge contains microfluidic channels that transport processed sample to five separate chambers where the qPCR occurs. Each chamber contains five channels with different sets of primers, allele-specific unlabeled mediator probes, and universal fluorescent reporters. Three chambers amplify targets for either *IDH1* R132, *IDH2* R140, or *IDH2* R172 codons and the remaining two chambers amplify only the sample processing control (SPC). The SPC, a conserved fragment in the *K1F11* control gene, is in the fifth channel of each qPCR chamber and is used as a process control, to measure the amount of amplifiable DNA in the sample, and to compare against mutations detected by the assay.

When the qPCR cycles are completed, the system reviews PCR curve data for the SPC and calculates a cycle of quantification (Cq) value. This is repeated for PCR curves generated by the target signals, and a delta Cq (ΔCq) value is calculated between the SPC and the target alteration. The automated report results whether an alteration was detected within a codon, the Cq of the target alteration curve, the ΔCq between the target alteration and the SPC within the same chamber, and the mean Cq of all SPCs (SPC_x̅_ Cq). Per manufacturer, optimal input is > 500 ng extracted DNA (50 ul extracted DNA with ≥ 10 ng/ul DNA concentration) or 1–5 FFPE sections with  ≥  10% neoplastic content.

Cartridge output was analyzed using software version v4.5.0.764 on the Idylla console and software version 4.2.3 of R Statistical Software. Per manufacturer, SPC_x̅_ Cq > 35 indicates low DNA input or quality. An invalid result is reported for a codon when its corresponding control is not detected, and if more than one control is not detected, the entire cartridge result is invalid. Acceptable ΔCq between control Cq and codon Cq for *IDH1* R132, *IDH2* R140, and *IDH2* R172K are ≤ 10, ≤8, and ≤ 7, respectively. To mimic current clinical testing using the Idylla *EGFR* and *KRAS* assays, we used five FFPE sections for biopsies or cases with scant tissue and one FFPE section for resections with abundant tissue.

## Results

### Accuracy in clinical samples

**Fig. 1:**
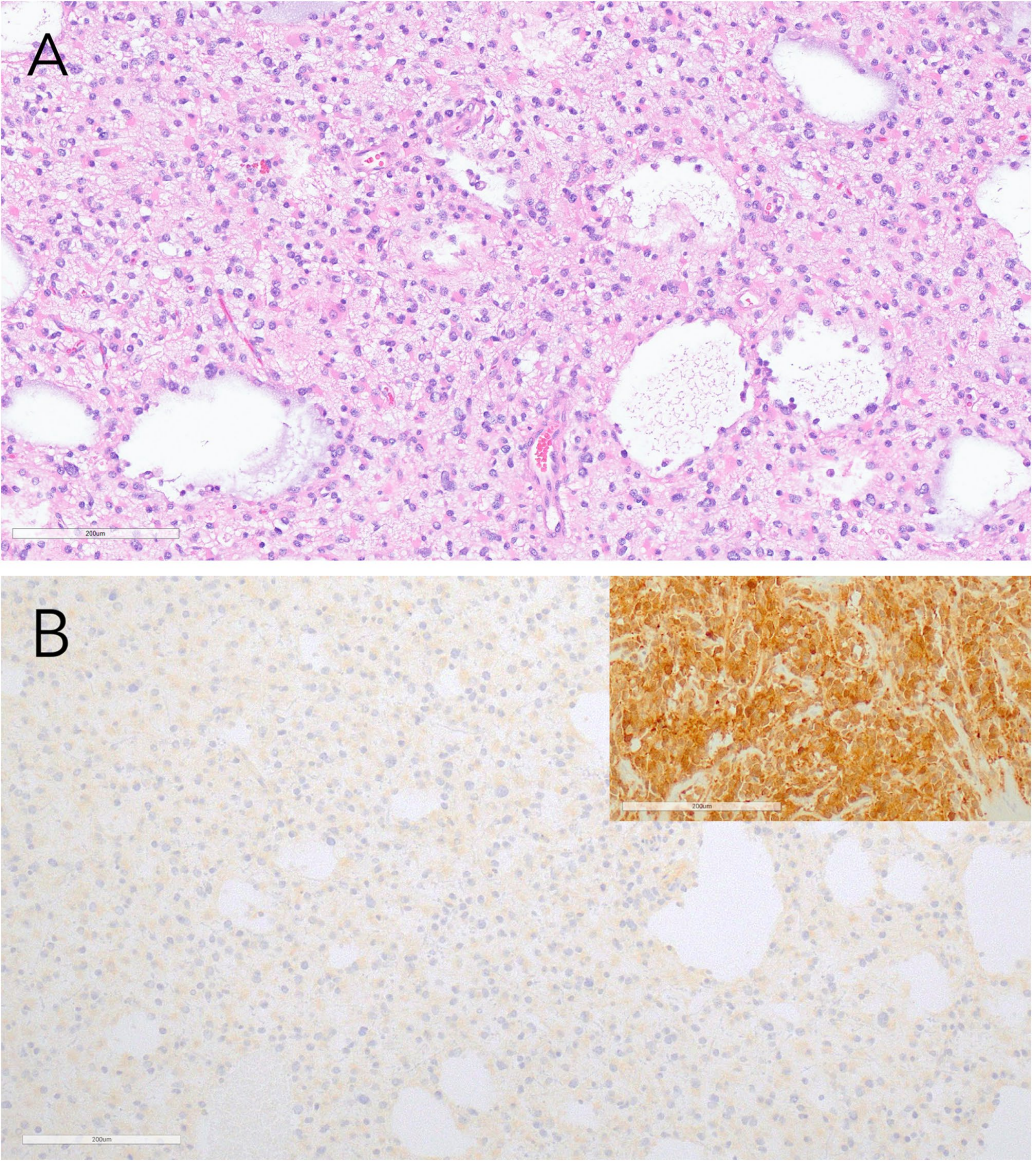
The H&E and *IDH1* R132H IHC sections are shown for case 20. Morphology and other immunohistochemical findings raised the clinical suspicion of an *IDH1/2* alteration (**A**). However, IHC for IDH1 R132H was negative, demonstrating only a background non-specific blush interpreted as negative (**B**). The positive control is shown in the inset for reference. NGS and Idylla testing revealed *IDH1* p.R132C alteration

Thirty-one infiltrating glioma FFPE tissue specimens were tested, all of which had known *IDH*1/2 status as determined by the Oncomine Comprehensive Assay v2 performed as part of routine clinical testing. Neoplastic content ranged from 10 to 90%, and variant allele fraction of the known *IDH1/2* mutation ranged from 21 to 56.6%. Two of the specimens were previously frozen sections for intraoperative analysis that were subsequently FFPE. Specific hotspot alterations tested included *IDH1* p.R132H (*n* = 15), *IDH1* p.R132G (*n* = 2), *IDH1* p.R132L (*n* = 1), *IDH1* p.R132S (*n* = 2), *IDH1* p.R132C (*n* = 1), *IDH2* p.R172K (*n* = 1), *IDH2* p.R172M (*n* = 1). Seven cases were IDH wild-type, and one case had a *IDH1* p.I117T variant of uncertain significance. All cases with *IDH1* p.R132H were positive by IDH1 R132H IHC, while the remaining cases were negative by IHC (Fig. 1). The mean SPC_x̅_ Cq for *IDH1* p.R132 and *IDH2* p.R172 alterations was 33.9 (SD 1.7) and 32.8 (SD 1.5), respectively, and the mean target Cq for *IDH1* p.R132 and *IDH2* p.R172 samples were 38.0 (SD 1.6) and 35.5 (SD 3.3), respectively. Specific cases and Cq values are shown in Table 1. To evaluate other alterations on the assay additional non-glioma specimens with *IDH1* p. R132L (*n* = 1), *IDH2* p.R140Q (*n* = 1), *IDH2* p.R172G (*n* = 3), and IDH2 p.R172W (*n* = 3) were also tested. Only one case with *IDH2* p.R172G was not detected by the assay, though the VAF was 2% and below the expected LOD.

**Table 1:** Accuracy studies on FFPE tissue slides of glioma cases. *Three cases had invalid results with FFPE tissue slides, all of which had successful repeat testing performed with DNA extracted from additional unstained FFPE slides (see text). **One discordant case (number 19) had an additional alteration, IDH2 R172K reported by Idylla but that was not detected by NGS. ^**†**^One case (number 34) was not detected by Idylla but had VAF below the limit of detection

Number	Year specimen collected and tumor type	Alteration	Variant allele frequency of IDH1/2 alteration by NGS	Idylla Result	SPCx̅	Cq target	ΔCq
1	2018 Glioma	IDH1_R132H	36.5%	IDH1_R132	32.1	36.9	4.8
2	2018 Glioma	IDH1_R132H	41.2%	IDH1_R132	37	40.1	4.3
3	2019 Glioma	IDH1_R132H	38.4%	IDH1_R132	32.3	37.4	5
4	2019 Glioma	IDH1_R132H	43.8%	IDH1_R132	33.8	38	4
5	2019 Glioma	IDH1_R132H	56.6%	Invalid*			
5-Repeat				IDH1_R132	33.5	38.4	4.8
6	2019 Glioma	IDH1_R132H	32.7%	IDH1_R132	34	38.7	4.2
7	2019 Glioma	IDH1_R132H	38.2%	IDH1_R132	34.7	40.1	5.3
8	2019 Glioma	IDH1_R132H	40.9%	IDH1_R132	33.8	38.9	5.1
9	2019 Glioma	IDH1_R132H	46.2%	IDH1_R132	32.8	37.9	5
10	2020 Glioma	IDH1_R132H	39.5%	IDH1_R132	30.2	34.6	4.4
11	2019 Glioma	IDH1_R132H	33.6%	Invalid*			
11-Repeat				IDH1_R132	36.8	39.1	3
12	2019 Glioma	IDH1_R132H	43.7%	IDH1_R132	31.9	36.2	4.2
13	2019 Glioma	IDH1_R132H	47.6%	IDH1_R132	33.6	38.2	4.3
14	2022 Glioma	IDH1_R132H	21%	IDH1_R132	32.1	36.7	4.6
15	2022 Glioma	IDH1_R132H	28.7%	IDH1_R132	37	40.4	2.6
16	2019 Glioma	IDH1_R132G	45.5%	IDH1_R132	35.1	38.3	1.8
17	2022 Glioma	IDH1_R132G	40.3%	IDH1_R132	36.1	38.6	2.1
18	2020 Glioma	IDH1_R132S	33.7%	IDH1_R132	33.5	36.7	3.2
19	2019 Glioma	IDH1_R132S	41.4%	IDH1_R132	34	37.1	2.2
				IDH2_R172**		36.7	3
20	2022 Glioma	IDH1_R132C	43.1%	IDH1_R132	33.9	38.8	4.9
21	2018 Glioma	IDH1_I117T	40.3%	No mutation	30.9		
22	2019 Glioma	IDH2_R172K	48.9%	IDH2_R172	31.9	31.7	0
23	2023 Glioma	IDH2_R172M	29%	IDH2_R172	31.7	32.7	1.3
24	2019 Glioma	No mutation	N/A	No mutation	32.8		
25	2019 Glioma	No mutation	N/A	No mutation	37.4		
26	2019 Glioma	No mutation	N/A	No mutation	35.8		
27	2019 Glioma	No mutation	N/A	Invalid*			
27 - Repeat				No mutation	37.7		
28	2019 Glioma	No mutation	N/A	No mutation	37.9		
29	2022 Glioma	No mutation	N/A	No mutation	29.3		
30	2022 Glioma	No mutation	N/A	No mutation	32.3		
31	2024 Glioma	IDH1_R132L	5%	IDH1_R132	33.9	39.3	5.4
32	2023 Adenocarcinoma, liver	IDH1_R132L	8%	IDH1_R132	34.4	41.2	6.8
33	2023 Acute myeloid leukemia, bone marrow	IDH2_R140Q	72%	IDH1_R140	31.2	33.7	2.5
34	2024 EBV-associated lymphoproliferative neoplasm, lymph node	IDH2_R172G	2%	**No mutation** ^**†**^	32.3	N/A	N/A
35	2023 Cholangiocarcinoma, liver	IDH2_R172G	14%	IDH2_R172	34.4	39.2	5.7
36	2023 Carcinoma, soft tissue	IDH2_R172G	27%	IDH2_R172	30.5	33.8	3.4
37	2023 Cholangiocarcinoma, liver	IDH2_R172W	21%	IDH2_R172	34.9	37.7	2.8
38	2023 Cholangiocarcinoma, liver	IDH2_R172W	25%	IDH2_R172	33.3	38.9	5.6
39	2022 Adenocarcinoma, liver	IDH2_R172W	33%	IDH2_R172	33.3	35.3	1.1

Compared to the Oncomine Comprehensive Assay v2 targeted NGS panel, concordance in detection using FFPE slides was 97% (38/39) due to a presumably false positive *IDH2* p.R172K (SPC_x̅_ Cq 34 and target Cq 36.7) by Idylla in addition to the *IDH1* p.R132H alteration (target Cq 37.1) that was detected by both NGS and Idylla in case number 19. This *IDH2* p.R172K alteration was not detected when retested with extracted DNA (832 ng) from serially following sections instead of scraped FFPE tissue. There was not enough remaining tissue to retest with scraped FFPE tissue.

Moreover, of the thirty-nine samples tested, three samples initially failed when using FFPE tissue sections. Invalid results in two FFPE specimens that were previously frozen for intraoperative consultation *(*case 11: with *IDH1* R132H with 33.6% VAF by NGS and case 27 with wild-type *IDH1/2* with > 60% neoplastic content by pathologist estimation) were likely due to insufficient or poor**-**quality DNA input. Since no additional slides were available, repeat testing was performed with 50 ng of previously extracted DNA from NGS testing, leading to successful, concordant results (SPC_x̅_ Cq 36.8 and *IDH1* p.R132 Cq of 39.1 in case 11). For the third case with invalid results, (case 5 with *IDH1* R132H and 56.6% VAF ) DNA extraction of a deeper FFPE section yielded 764 ng DNA, which was above the minimum recommended input of 500 ng. Repeat testing using this extracted DNA resulted in concordant results (SPC_x̅_ Cq of 33.5 and *IDH1* p.R132 Cq of 38.4). Including these three cases where extracted DNA was used to rescue the invalid results obtained from the FFPE section, overall concordance was 97% (38/39). All three of these specimens were from 2019 and were not significantly older than the samples that were successful on first attempt.

### Limit of detection, limit of input, and reproducibility studies

Extracted DNA from Horizon DNA controls HD677 (*IDH1* p.R132H) and HD680 (*IDH2* p.R172K) that were diluted into HD678/HD681 (wild-type DNA) were successfully tested in triplicate with 50 ng input at VAF of 7% and 5% (Supplementary Table 2). Given limited availability of cartridges, we further tested at 2.5% VAF in a step-wise manner at lower DNA inputs (50 ng, then 10 ng, and 20 ng). LOD was established for HD680 repeated in triplicate with 10 ng input at VAF 2.5% (mean SPC_x̅_ Cq 35.0 (SD 0.23), mean *IDH2* p.R172 Cq 39.3 (SD 0.40)), and for HD677 with 20 ng input at VAF 2.5% (mean SPC_x̅_ Cq 33.7 (SD 0.20), mean *IDH1* p.R132 Cq 39.5 (SD 0.79)).

Extracted DNA from two FFPE glioma samples, *IDH1* p.R132H with VAF of 39.5% and *IDH2* p.R172K with VAF 48.9% (Sample 22), were diluted with DNA extracted from *IDH1/2* wild-type FFPE glioma sample (Supplementary Table 3). Given limited availability of cartridges, we first tested at 10% VAF with 50 ng and then tested in duplicate at both 5% VAF with 50 ng input and 2.5% VAF with 50 ng input. LOD was established for *IDH2* p.R172K repeated in triplicate with 50 ng input at VAF 2.5% (mean SPC_x̅_ Cq 34.0 (SD 0.40), mean *IDH2* p.R172 Cq 39.6 (SD 0.50)), and for *IDH1* p.R132H with 50 ng input at VAF 5% (mean SPC_x̅_ Cq 33.2 (SD 0.10), mean *IDH1* p.R132 Cq 39.5 (SD 0.60)).

### Estimation of VAF using ΔCq values

Evaluation of ΔCq versus variant allele frequency in FFPE samples with *IDH1* p.R132H alterations, HD677, and HD680 did not demonstrate strong correlations (Supplementary Fig. 1), precluding use of this assay for semi-quantitation. Input amount varied for the FFPE clinical samples, and as expected the R^2^ value (0.14) was noticeably lower when compared to HD677 and HD680 controls at 50 ng input (0.516 and 0.453, respectively).

### Reproducibility studies in clinical samples

Reproducibility studies were performed using two FFPE glioma samples, cases 8 and 22, with *IDH1* p.R132H and *IDH2* p.R172K mutations respectively, by testing FFPE sections on three separate days (Supplementary Table 5). Complete agreement was observed in both samples: *IDH1* p.R132H (40.9% VAF, 1 FFPE section with $\sim$932 ng input DNA, mean SPC_x̅_ Cq 33.8 (SD 0.26), mean* IDH1* p.R132 Cq 38.9 (SD 0.12)) and *IDH2* p.R172K (48.9% VAF, 1 FFPE section with $\sim$2,616 ng input DNA, mean SPC_x̅_ Cq 32 (SD 0.56), mean *IDH2* p.R172 Cq 31.4 (SD 1.08)).

### Comparison of assay performance using scraped FFPE tissue versus DNA extracted from FFPE tissue

Given the initial failure of FFPE in three samples with subsequent success on extracted DNA, we tested four additional FFPE glioma samples by both using FFPE tissue sections scraped directly into the cartridge and by using DNA extracted from an equivalent number of FFPE tissue sections. The SPC_x̅_ Cqs and target codon Cqs did not vary significantly between scraped FFPE tissue from slides and extracted DNA from FFPE tissue sections (Table 2). The amount of DNA extracted ranged from 932 ng to 2616 ng when using 1 section or 832 ng when using 5 sections for a small biopsy.

**Table 2:** Performance of assay on extracted DNA from FFPE tissue versus tissue from scraped FFPE sections

Sample	Year specimen collected	Alteration	Sample type	VAF	SPCx̅ Cq	Cq target	ΔCq
8	2018	*IDH1* p.R132H	FFPE (1 section)	40.9%	33.8	38.9	5.1
8	2018	*IDH1* p.R132H	DNA 932 ng	40.9%	34.6	39.2	4.5
22	2019	*IDH2* p.R172K	FFPE (1 section)	48.9%	31.9	31.7	0
22	2019	*IDH2* p.R172K	DNA 2616 ng	48.9%	31.8	30.6	-1.3
19	2019	*IDH1* p.R132S	FFPE (5 sections)	41.4%	34	37.1	2.2
19	2019	*IDH1* p.R132S	DNA 832 ng	41.4%	35.5	38	2.5
30	2022	Wild-type	FFPE (1 section)	N/A	32.3	N/A	N/A
30	2022	Wild-type	DNA 1172 ng	N/A	31.0	N/A	N/A

### Comparison of assay performance using extracted DNA from FFPE tissue vs. peripheral blood

To compare FFPE performance with other sample types and to assess the performance of the assay on *IDH2* p.R140 detection, extracted DNA from the peripheral blood of three patients with hematologic malignancy (*IDH1* p.R132H at VAF 46%, *IDH2* p.140Q at 50% VAF, and wild-type) were tested at 200 ng, 100 ng, and 50 ng input in duplicate (Supplementary Table 4). All eighteen samples were concordant with results from ddPCR and had lower SPC_x̅_ Cq and target Cq values compared to FFPE tissue samples even with lower DNA inputs.

## Discussion

*IDH1* and *IDH2* mutational status is a critical biomarker in the evaluation of glioma. While immunohistochemistry and next-generation sequencing are well-established methods for determining *IDH1/2* mutational status, each methodology suffers from drawbacks. The benefits of immunohistochemistry are that it requires only a single slide, has a rapid TAT, and can be evaluated by the surgical pathologist as part of immunohistochemical workup, but the most commonly used antibody clone can only detect the *IDH1* p.R132H alteration [7]. Next-generation sequencing methods are more comprehensive and can detect any oncogenic missense alteration in *IDH1* or *IDH2* [8], but sequencing requires a high DNA input and the TAT can be over two weeks. Therefore, a method that combines benefits of both assays -- rapid TAT, minimal material, and ability to detect any alteration in *IDH1/2* -- would be ideal.

Rapid TAT of *IDH1/2* mutational status would allow for quicker treatment planning and initiation, as well as more efficient enrollment in clinical trials. Clinical trials often have very limited windows for enrollment, and batched tests or high complexity tests such as NGS have TATs that can make it difficult for clinicians to enroll their patients in time. Other assay possibilities have been explored previously. For example, recent studies demonstrated the clinical utility of quantitative PCR and digital droplet PCR tests for determining *IDH1/IDH2* mutational status that had good concordance with IHC and NGS [9, 10]. Digital droplet PCR (ddPCR) is a technique where a water-oil emulsion partitions the sample into many individual reactions so that either zero, one, or more target molecules are present in each independent reaction. This approach can detect low levels of positives against a strong background of negatives. Thus, the technique can achieve limit of detection down to $\sim$0.1%, outperforming the standard real-time quantitative PCR (qPCR) in rare target detection. However, this theoretical sensitivity is limited in FFPE samples due to background formalin fixation artifacts that affect signal to noise ratio. Moreover, the high sensitivity of ddPCR comes at the cost of limited dynamic range and longer assay setup times. The dynamic range depends on the number of partitions analyzed and on most commercial systems this is typically around four orders of magnitude compared to seven orders of magnitude achievable by qPCR. In contrast to single cartridge use, ddPCR often requires batching of samples to maintain realistic cost per test and efficient use of technologist time, which can affect TAT. Finally, though glioma samples often have limited tissue, they have adequate tumor cellularity and do not require the high sensitivity attainable by ddPCR.

Here, we use the Idylla Rapid *IDH1/2* Mutation Assay, a single sample cartridge-based, fully automated real-time PCR assay that can detect a variety of mutations in *IDH1* and *IDH2*. Hands-on technologist time is approximately five minutes for initial set-up of the assay, and the Idylla cartridge and console system performs automated DNA extraction, real-time PCR, result interpretation, and report generation in ninety-five minutes. Input for the assay can consist of either direct sample or extracted DNA from FFPE for glioma patients and direct sample or extracted DNA from blood and bone marrow in EDTA for acute myeloid leukemia patients.

The Idylla Rapid *IDH1/2* Mutation Assay had 97% concordance with NGS, with failure in one case due to a non-specific call using scraped FFPE tissue that was not seen when repeated with extracted DNA. Notably, there were no false negatives in cases with sufficient material. The assay performed well even with low DNA input (50 ng), and LOD ranged from 2.5 to 5% with increased sensitivity for *IDH2* R172K compared to *IDH1* p.R132H using both inputs of genomic DNA from controls as well as DNA from FFPE samples. Notably, for *IDH1* p.R132H, the LOD for FFPE samples was 5% VAF with 50 ng input compared to 2.5% VAF with 20 ng input using genomic DNA. However this LOD should still be sufficient for FFPE glioma samples which tend to have high tumor cellularity.

One limitation of the study is that some of the *IDH1/2* alterations covered by the assay are extremely rare. No standard control material was commercially available, and no clinical samples were available at our institution, precluding definitive assessment of these rare variants.

Some considerations with clinical implementation of the assay are that it may be sensitive to pre-analytic conditions that affect the quality of the DNA. In our experiments, specimens that had previously undergone frozen section analysis for intraoperative diagnosis led to invalid results in two cases. Using DNA that was separately extracted from slides cut from previously frozen tissue blocks were successful. Similarly in one case with a false positive *IDH2* p.R172K detection, repeat testing using DNA separately extracted from FFPE slides did not show this alteration. This may indicate an issue with pre-PCR sample processing of FFPE in the Idylla cartridge, perhaps due to PCR inhibition such as from reagents using during preparation of frozen tissue, like OCT Tissue Tek, that are not removed during the Idylla nucleic acid extraction process. In scenarios, like at our institution, where these samples usually undergo additional testing by a large cancer gene panel, this could be added as a disclaimer to the test and would not preclude routine use for initial rapid testing.

Overall, the Idylla extraction method is a reliable assay to rapidly and accurately determine *IDH1/2* status from unstained slides in a few hours. While the promise of same-day *IDH1/2* status is attractive, it should be noted that other upstream factors can delay TAT from the oncologist’s perspective. Therefore, thoughtful consideration of laboratory workflows and assay implementation are important.

### Electronic supplementary material

Below is the link to the electronic supplementary material.

Supplementary Material 1

## Data Availability

All data used in this manuscript is provided within the manuscript and the supplementary information files.

## Abbreviations

ddPCR: droplet digital PCR
FFPE: formalin-fixed paraffin-embedded
IHC: immunohistochemistry
NGS: next-generation sequencing
VAF: variant allele fraction

## References

[1] Molenaar RJ (2018). Wild-type and mutated IDH1/2 enzymes and therapy responses. Oncogene.

[2] Han S (2020). IDH mutation in glioma: molecular mechanisms and potential therapeutic targets. Br J Cancer.

[3] Kaminska B et al. Consequences of IDH1/2 mutations in Gliomas and an Assessment of inhibitors targeting mutated IDH proteins. Molecules, 2019. 24(5). 10.3390/molecules24050968 PMC6429355 30857299

[4] Yan H (2009). IDH1 and IDH2 mutations in gliomas. N Engl J Med.

[5] Mellinghoff IK (2023). Vorasidenib in IDH1- or IDH2-Mutant low-Grade Glioma. N Engl J Med.

[6] Slocum CC (2022). Towards a single-assay approach: a combined DNA/RNA sequencing panel eliminates diagnostic redundancy and detects clinically-relevant fusions in neuropathology. Acta Neuropathol Commun.

[7] Agarwal S (2013). Comparative study of IDH1 mutations in gliomas by immunohistochemistry and DNA sequencing. Neuro Oncol.

[8] Śledzińska P (2022). Glioma 2021 WHO classification: the superiority of NGS over IHC in Routine Diagnostics. Mol Diagn Ther.

[9] Nelson EJ (2023). Clinical evaluation of IDH Mutation Status in Formalin-fixed paraffin-embedded tissue in Gliomas. Mol Diagn Ther.

[10] Wang J (2015). IDH1 mutation detection by droplet digital PCR in glioma. Oncotarget.

