# Supplementary material for: Evaluation of the rapid Idylla IDH1-2 mutation assay in FFPE glioma samples
Source: Diagn Pathol. 2024 May 25;19:70. doi: 10.1186/s13000-024-01492-3 (PMC11128120; doi:10.1186/s13000-024-01492-3)
Supplement: Supplementary file 1 — Supplementary Material 1 [file 13000_2024_1492_MOESM1_ESM.docx]

**Supplementary Figure 1.** The ΔCq was plotted against variant allele frequency for all clinical FFPE samples with the IDH1 p.R132H alteration and the controls for *IDH1* p.R132H (Horizon 677) and *IDH2* p.R172K (Horizon 680).


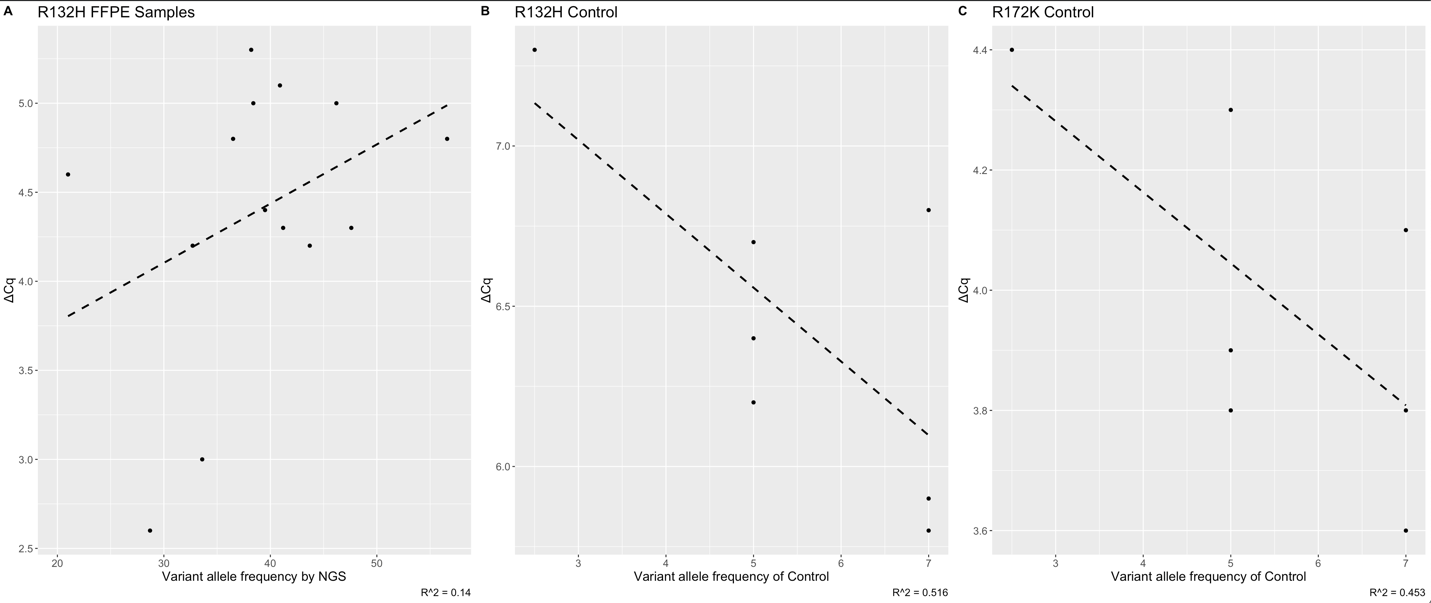


**Supplementary Table 1.** The assay detects five alterations in *IDH1* codon 132, four alterations in *IDH2* codon 140, and six alterations in *IDH2* codon 172. The specific protein change and cDNA changes are listed below.

| *IDH1* mutations | | |
| --- | --- | --- |
| Codon 132 | p.R132C | c.394C>T |
|  | p.R132G | c.394C>G |
|  | p.R132H | c.395G>A |
|  | p.R132L | c.395G>T |
|  | p.R132S | c.394C>A |
| *IDH2* mutations | | |
| Codon 140 | p.R140G | c.418C>G |
|  | p.R140L | c.419G>T |
|  | p.R140Q | c.419G>A |
|  | p.R140W | c.418C>T |
| Codon 172 | p.R172K | c.515G>A |
|  | p.R172G | c.514A>G |
|  | p.R172M | c.515G>T |
|  | p.R172S | c.516G>T |
|  | p.R172S | c.516G>C |
|  | p.R172W | c.514A>T |

**Supplementary Table 2.**  Limit of detection studies and reproducibility studies using Horizon control genomic DNA. The mean and standard deviation (SD) are calculated from the repeats, if available.

| **7%, 50 ng** | | | | |
| --- | --- | --- | --- | --- |
| *IDH1* p.R132H | DAY 1, TECH 1 | DAY 2. TECH  2 | DAY 3, TECH 1 | MEAN (SD) |
| SPCx̅ Cq | 32.2 | 32.2 | 32.5 | 32.3 (0.17) |
| Cq *IDH1* p.R132 | 38.3 | 39.1 | 38.4 | 38.6 (0.44) |
| ΔCq | 5.8 | 6.8 | 5.9 | 6.2 (0.55) |
| *IDH2* p.R172K | DAY 1, TECH 1 | DAY 2. TECH  2 | DAY 3, TECH 1 | MEAN (SD) |
| SPCx̅ Cq | 32.8 | 32.5 | 32.5 | 32.6 (0.17) |
| Cq *IDH2* p.R172 | 36.5 | 36.1 | 36.0 | 36.2 (0.26) |
| ΔCq | 4.1 | 3.8 | 3.6 | 3.8 (0.25) |
| **5%, 50 ng** | | | | |
| *IDH1* R132H | DAY 1, TECH 1 | DAY 2. TECH  2 | DAY 3, TECH 1 | MEAN (SD) |
| SPCx̅ Cq | 32.1 | 32.4 | 32.3 | 32.3 (0.15) |
| Cq *IDH1* p.R132 | 38.4 | 38.8 | 39.4 | 38.9 (0.50) |
| ΔCq | 6.2 | 6.4 | 6.7 | 6.4 (0.25) |
| *IDH2* p.R172K | DAY 1, TECH 1 | DAY 2. TECH  2 | DAY 3, TECH 1 | MEAN (SD) |
| SPCx̅ Cq | 32.6 | 32.5 | 32.8 | 32.6 (0.15) |
| Cq *IDH2* p.R172 | 36.3 | 36.3 | 36.4 | 36.3 (0.06) |
| ΔCq | 3.8 | 4.3 | 3.9 | 4.0 (0.26) |
| **2.5%, 50 ng** | | | | |
| *IDH1* p.R132H | DAY 1, TECH 1 |  |  |  |
| SPCx̅ Cq | 32.6 |  |  |  |
| Cq *IDH1* p.R132 | 40.3 |  |  |  |
| ΔCq | 7.3 |  |  |  |
| *IDH2* p.R172K | DAY 1, TECH 1 |  |  |  |
| SPCx̅ Cq | 32.6 |  |  |  |
| Cq *IDH2* p.R172 | 36.9 |  |  |  |
| ΔCq | 4.4 |  |  |  |
| **2.5%, 20 ng** | | | | |
| *IDH1* p.R132H | DAY 1, TECH 1 | DAY 2. TECH  2 | DAY 3, TECH 1 | MEAN (SD) |
| SPCx̅ Cq | 33.5 | 33.7 | 33.9 | 33.7 (0.20) |
| Cq *IDH1* p.R132 | 39.8 | 40.1 | 38.6 | 39.5 (0.79) |
| ΔCq | 6.5 | 6.6 | 5.2 | 6.1 (0.78) |
| **2.5%, 10 ng** | | | | |
| *IDH1* p.R132H | DAY 1, TECH 1 |  |  |  |
| SPCx̅ Cq | 34.7 |  |  |  |
| Cq *IDH1* p.R132 | Not detected |  |  |  |
| ΔCq | N/A |  |  |  |
| *IDH2* p.R172K | DAY 1, TECH 1 | DAY 2. TECH  2 | DAY 3, TECH 1 | MEAN (SD) |
| SPCx̅ Cq | 34.9 | 34.9 | 35.3 | 35.0 (0.23) |
| Cq *IDH2* p.R172 | 38.9 | 39.7 | 39.3 | 39.3 (0.40) |
| ΔCq | 4.9 | 4.8 | 4 | 4.6 (0.49) |

**Supplementary Table 3.**  Limit of detection studies and reproducibility studies on DNA extracted from two FFPE samples, *IDH1* p.R132H with VAF 39.5% and *IDH2* p.R172K with VAF 48.9%, were performed by dilution using DNA extracted from an *IDH1/2* wild-type FFPE sample. The mean and standard deviation (SD) are calculated from the repeats, if available.

| **10%, 50 ng** | | | | |
| --- | --- | --- | --- | --- |
| *IDH1* p.R132H | DAY 1, TECH 1 |  |  |  |
| SPCx̅ Cq | 33.4 |  |  |  |
| Cq *IDH1* p.R132 | 39.2 |  |  |  |
| ΔCq | 5.7 |  |  |  |
| *IDH2* p.R172K | DAY 1, TECH 1 |  |  |  |
| SPCx̅ Cq | 31.7 |  |  |  |
| Cq *IDH2* p.R172 | 34.8 |  |  |  |
| ΔCq | 3.3 |  |  |  |
| **5%, 50 ng** | | | | |
| *IDH1* p.R132H | DAY 1, TECH 1 | DAY 2. TECH  2 | DAY 3, TECH 1 | MEAN (SD) |
| SPCx̅ Cq | 33.3 | 33.2 | 33.1 | 33.2(0.10) |
| Cq *IDH1* p.R132 | 39.5 | 40.1 | 38.9 | 39.5(0.60) |
| ΔCq | 6.2 | 6.7 | 5.7 | 6.2(0.50) |
| *IDH2* p.R172K | DAY 1, TECH 1 | DAY 2. TECH  2 |  | MEAN (CV) |
| SPCx̅ Cq | 33.7 | 33.8 |  | 33.8(0.71) |
| Cq *IDH2* p.R172 | 38.2 | 37.8 |  | 38.0(0.28) |
| ΔCq | 5.1 | 3.9 |  | 4.5(0.85) |
| **2.5%, 50 ng** | | | | |
| *IDH1* p.R132H | DAY 1, TECH 1 | DAY 2. TECH  2 |  | MEAN (SD) |
| SPCx̅ Cq | 33.5 | 33.4 |  | 33.4 (0.07) |
| Cq *IDH1* p.R132 | 40.1 | Not detected |  |  |
| ΔCq | 6.6 | N/A |  |  |
| *IDH2* p.R172K | DAY 1, TECH 1 | DAY 2. TECH  2 | DAY 3, TECH 1 | MEAN (SD) |
| SPCx̅ Cq | 33.5 | 34.2 | 34.1 | 34.0(0.40) |
| Cq *IDH2* p.R172 | 39.1 | 40.1 | 39.5 | 39.6(0.50) |
| ΔCq | 5.0 | 5.4 | 5.4 | 5.3(0.23) |

**Supplementary Table 4.** Performance of assay on extracted DNA from peripheral blood hematologic malignancy samples. The variant allele frequencies were 46% and 50% for the *IDH1* p.R132H and *IDH2* p.R140Q samples, respectively.

| **200 ng** | | | |
| --- | --- | --- | --- |
| ***IDH1* p.R132H** | DAY 1, TECH 1 | DAY 2. TECH  2 | MEAN (SD) |
| SPCx̅ Cq | 29.7 | 29 | 29.4 (0.49) |
| Cq *IDH1* p.R132 | 33.5 | 32.9 | 33.2 (0.42) |
| ΔCq | 3.8 | 4 | 3.9 (0.14) |
| ***IDH2* p.R140Q** | DAY 1, TECH 1 | DAY 2. TECH  2 | MEAN (SD) |
| SPCx̅ Cq | 30.6 | 30.9 | 30.8 (0.21) |
| Cq *IDH2* p.R140 | 29.8 | 29.5 | 29.7 (0.21) |
| ΔCq | -1 | -1.6 | -1.3 (0.42) |
| ***IDH1/IDH2* wild-type** | DAY 1, TECH 1 | DAY 2. TECH  2 | MEAN (SD) |
| SPCx̅ Cq | 30.9 | 30.7 | 30.8 (0.14) |
| Target Cq | N/A | N/A | N/A |
| ΔCq | N/A | N/A | N/A |
| **100 ng** | | | |
| ***IDH1 p.*R132H** | DAY 1, TECH 1 | DAY 2. TECH  2 | MEAN (SD) |
| SPCx̅ Cq | 30.8 | 30.5 | 30.7 (0.21) |
| Cq *IDH1* p.R132 | 34.5 | 34.4 | 34.5 (0.07) |
| ΔCq | 3.6 | 3.9 | 3.75 (0.21) |
| ***IDH2* p.R172Q** | DAY 1, TECH 1 | DAY 2. TECH  2 | MEAN (SD) |
| SPCx̅ Cq | 32.2 | 32 | 32.1 (0.14) |
| Cq *IDH2* p.R172 | 30.5 | 30.3 | 30.4 (0.14) |
| ΔCq | -2.2 | -2 | -2.1 (0.14) |
| ***IDH1/IDH2* wild-type** | DAY 1, TECH 1 | DAY 2. TECH  2 | MEAN (SD) |
| SPCx̅ Cq | 32.2 | 31.9 | 32.1 (0.21) |
| Target Cq | N/A | N/A | N/A |
| ΔCq | N/A | N/A | N/A |
| **50 ng** | | | |
| ***IDH1 p.*R132H** | DAY 1, TECH 1 | DAY 2. TECH  2 | MEAN (SD) |
| SPCx̅ Cq | 31.7 | 31.9 | 31.8 (0.14) |
| Cq target | 35.9 | 35.6 | 35.8 (0.21) |
| ΔCq | 4.0 | 3.7 | 3.9 (0.21) |
| ***IDH1 p.*R172Q** | DAY 1, TECH 1 | DAY 2. TECH  2 | MEAN (SD) |
| SPCx̅ Cq | 32.7 | 33.1 | 32.9 (0.28) |
| Cq target | 31.4 | 31.8 | 31.6 (0.28) |
| ΔCq | -2 | -1.3 | -1.65 (0.49) |
| ***IDH1/IDH2* wild-type** | DAY 1, TECH 1 | DAY 2. TECH  2 | MEAN (SD) |
| SPCx̅ Cq | 32.8 | 32.8 | 32.8 (0) |
| Cq target | N/A | N/A | N/A |
| ΔCq | N/A | N/A | N/A |

**Supplementary Table 5.** Reproducibility studies were performed by testing FFPE sections from clinical samples, case 8 (*IDH1* p.R132H, 40.9% VAF) and case 22 (*IDH2* p.R172K, 48.9% VAF) on three separate days by two different technologists.

| **Case 8** | | | | |
| --- | --- | --- | --- | --- |
| *IDH1* p.R132H | DAY 1, TECH 1 | DAY 2. TECH  2 | DAY 3, TECH 1 | MEAN (SD) |
| SPCx̅ Cq | 34.1 | 33.6 | 33.7 | 33.8 (0.26) |
| Cq *IDH1* p.R132 | 39 | 38.8 | 38.8 | 38.9 (0.12) |
| ΔCq | 4.9 | 4.8 | 4.7 | 4.8 (0.10) |
| **Case 22** | | | | |
| *IDH2* p.R172K | DAY 1, TECH 1 | DAY 2. TECH  2 | DAY 3, TECH 1 | MEAN (SD) |
| SPCx̅ Cq | 31.9 | 32.6 | 31.5 | 32 (0.56) |
| Cq *IDH2* p.R172 | 31.7 | 32.3 | 30.2 | 31.4 (1.08) |
| ΔCq | 0 | 0.1 | -1.2 | -0.4 (0.72) |
